# Supplementary material for: Detection of Circulating Tumor Cells Using the Attune NxT
Source: Int J Mol Sci. 2022 Dec 20;24(1):21. doi: 10.3390/ijms24010021 (PMC9820284; doi:10.3390/ijms24010021)
Supplement: Supplementary file 1 [file ijms-24-00021-s001.zip › ijms-2055429-supplementary.pdf]

Supplementary Figure S1

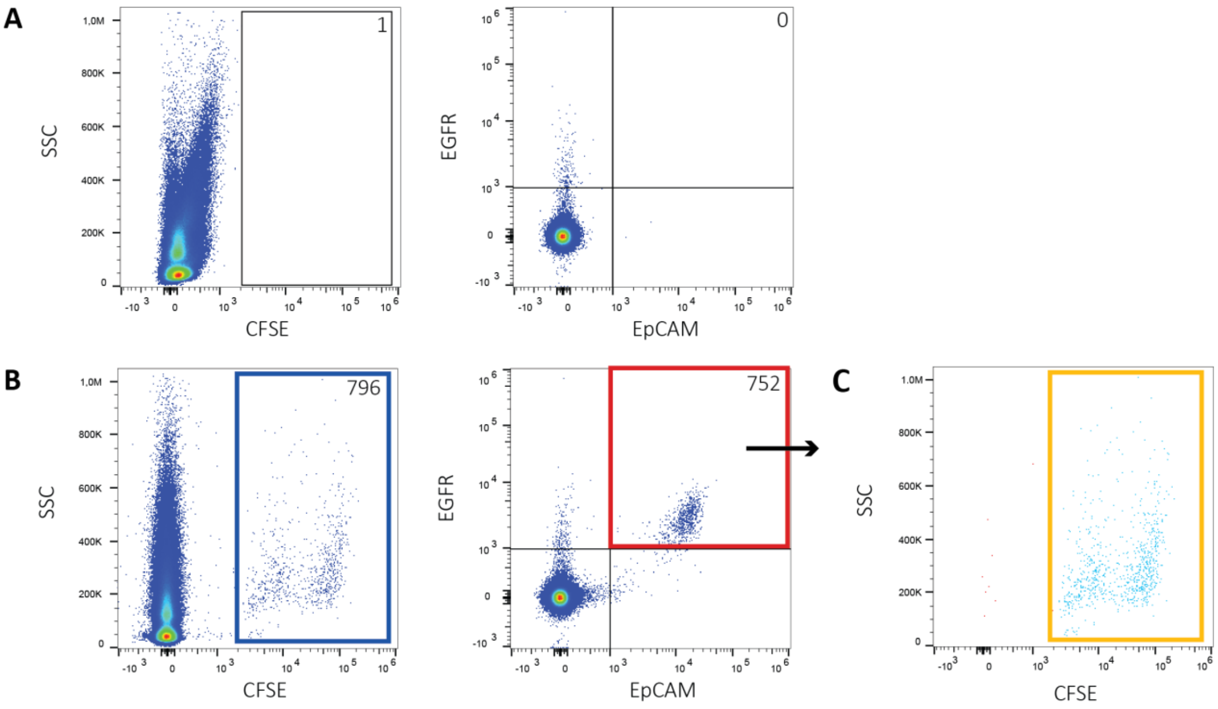

EpCAM and EGFR are suitable markers for CTC detection. Healthy donor blood was either (**A**) non-spiked or (**B**) spiked with 1,000 CFSE-labeled HCT116 cells. After PBMC isolation, samples were stained for EpCAM and EGFR, and analyzed using the Attune NxT. Tumor cells were detected by both CFSE staining (left column) and EpCAM + EGFR staining (right column). (**C**) Overlay of CFSE signal (blue) and EpCAM + EGFR signal (red). Blue square = CFSE+ events, represented as blue dots in (**C**), red square = EpCAM+EGFR+ events, represented as red dots in (**C**), yellow square = CFSE+EpCAM+EGFR+ events. Number of detected tumor cells is indicated in upper right corner. Representative figure of N=3.

Supplementary Figure S2

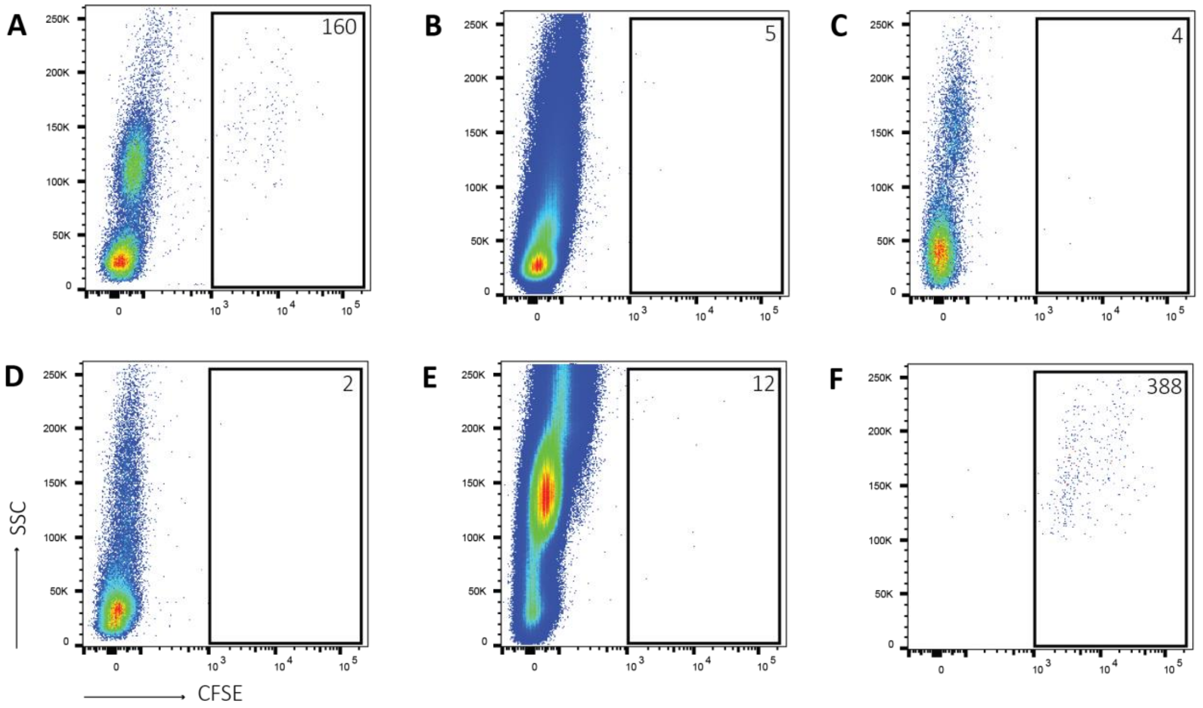

More than half of CTCs are lost during EpCAM enrichment. Healthy donor blood was spiked with 1,000 CFSE-labeled HT29 cells. After PBMC isolation, samples were enriched for EpCAM+ cells. Then, samples were stained for EpCAM and EGFR, and analyzed using the LSR Fortessa X20 (BD Biosciences). Tumor cells were detected by EpCAM + EGFR staining. CTC count in (**A**) the eluent of the PBMC fraction, (**B**) the flowthrough of the PBMC fraction, (**C**) the plasma fraction, (**D**) the Lymphoprep fraction and (**E**) the PMN fraction. (**F**) Eluent of 1,000 CFSE-labeled HT29 cells in medium. Number of detected tumor cells is indicated in upper right corner.

Supplementary Figure S3

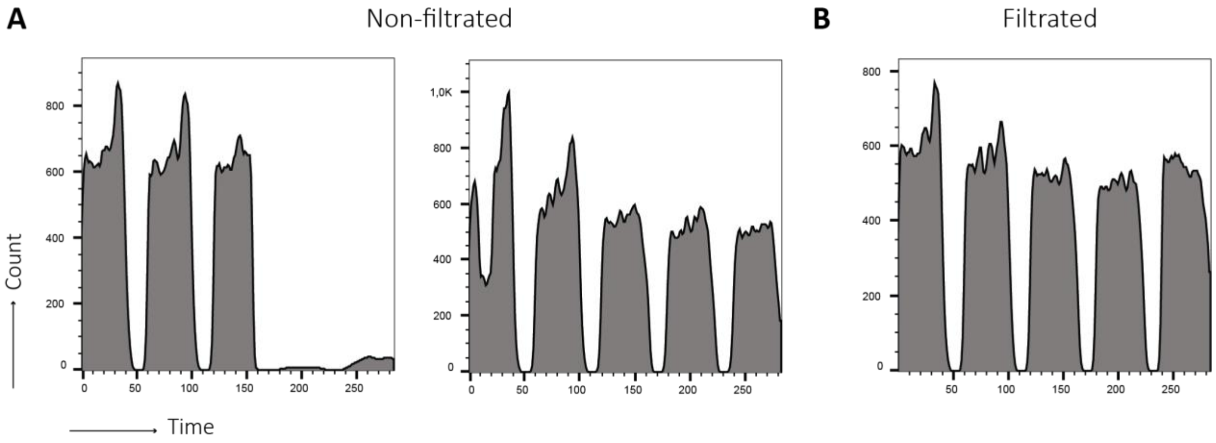

Sample filtration improves Attune NxT performance. (**A**) Analysis of non-filtrated samples results in either loss of (part of) a sample (left column) or irregular cell counts (right column). (**B**) Sample filtration provides stable cell counts. As the Attune NxT acquires samples in multiple phases, each peak represents the cell count in part of the sample over time.

Supplementary Figure S4

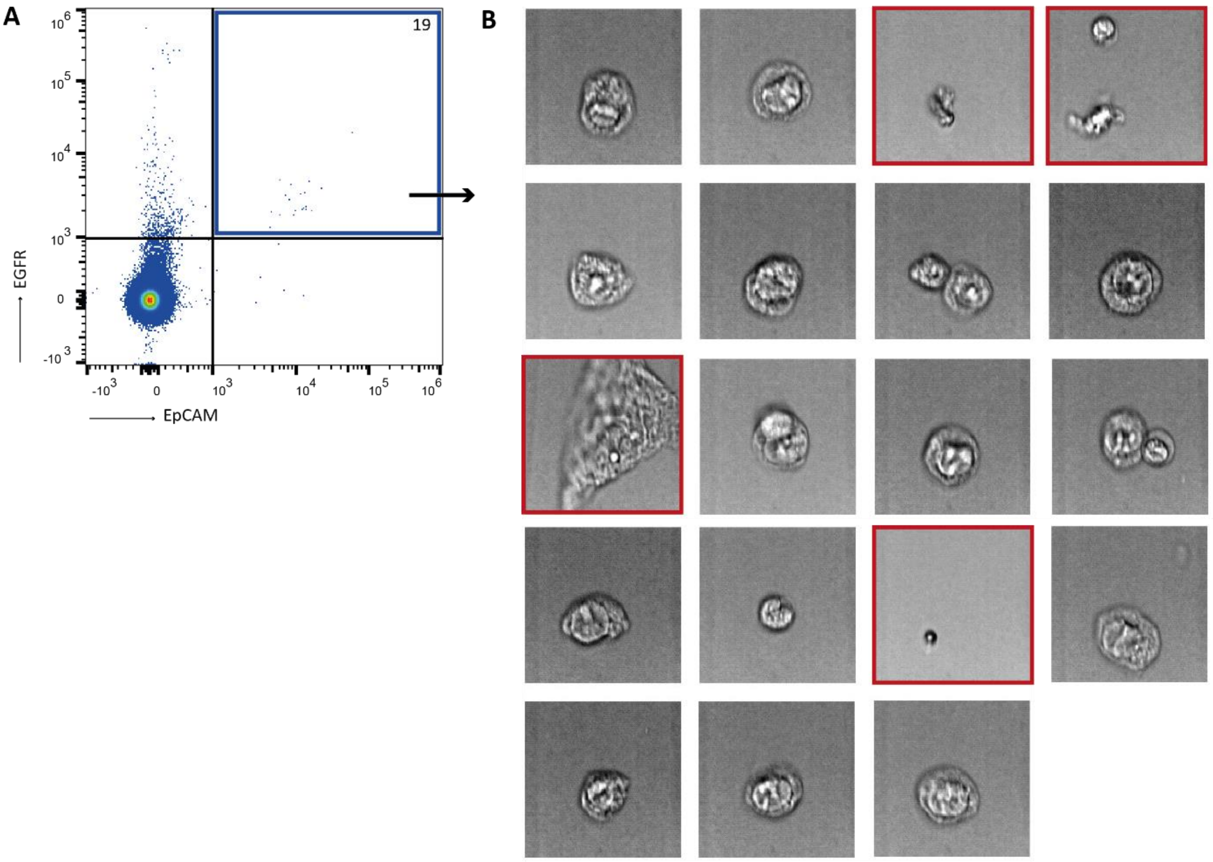

The majority of EpCAM+EGFR+ cells has a tumor cell-like morphology. Healthy donor blood was spiked with 10 HCT116 cells. After PBMC isolation, samples were stained for EpCAM and EGFR, and analyzed using the Attune CytPix. (**A**) Tumor cells were detected by EpCAM + EGFR staining. (**B**) EpCAM+EGFR+ cells have a tumor cell-like morphology. Red square = cell with no tumor cell-like morphology. Number of detected tumor cells is indicated in upper right corner. Representative figure of N=3.

Supplementary Figure S5

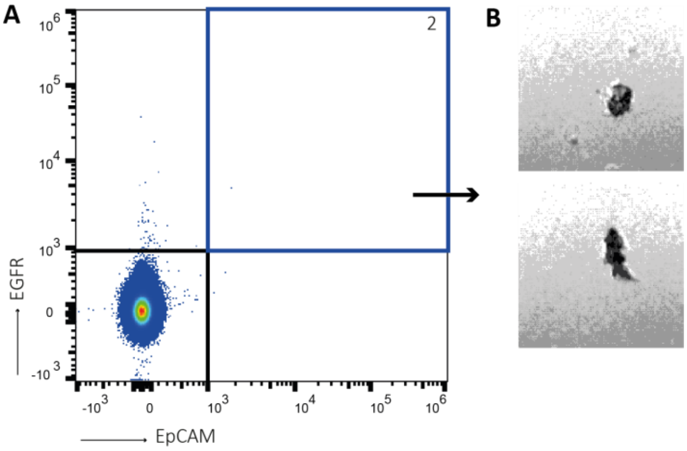

Healthy donor EpCAM+EGFR+ cells have no tumor cell-like morphology. Blood analysis of healthy donors. After PBMC isolation, samples were stained for EpCAM and EGFR, and analyzed using the Attune CytPix. (**A**) Possible tumor cells were detected by EpCAM + EGFR staining. (**B**) EpCAM+EGFR+ cells have no tumor cell-like morphology. Number of detected tumor cells is indicated in upper right corner. Representative figure of N=10.
